# Anti-NMDAR antibodies, the blood–brain barrier, and anti-NMDAR encephalitis

**DOI:** 10.3389/fneur.2023.1283511

**Published:** 2023-12-08

**Authors:** Xiarong Gong, Niya Wang, Hongyan Zhu, Ning Tang, Kunhua Wu, Qiang Meng

**Affiliations:** ^1^Faculty of Life Science and Technology, Kunming University of Science and Technology, Kunming, China; ^2^Department of MR, The First People's Hospital of Yunnan Province, Kunming, Yunnan, China; ^3^Department of Neurology, The First People's Hospital of Yunnan Province, Kunming, Yunnan, China; ^4^Department of Clinical Laboratory, The First People's Hospital of Yunnan Province, Kunming, Yunnan, China

**Keywords:** anti-NMDAR encephalitis, blood–brain barrier, neurovascular unit, NMDAR receptor, autoimmune disorder

## Abstract

Anti-N-methyl-D-aspartate receptor (anti-NMDAR) encephalitis is an antibody-related autoimmune encephalitis. It is characterized by the existence of antibodies against NMDAR, mainly against the GluN1 subunit, in cerebrospinal fluid (CSF). Recent research suggests that anti-NMDAR antibodies may reduce NMDAR levels in this disorder, compromising synaptic activity in the hippocampus. Although anti-NMDAR antibodies are used as diagnostic indicators, the origin of antibodies in the central nervous system (CNS) is unclear. The blood–brain barrier (BBB), which separates the brain from the peripheral circulatory system, is crucial for antibodies and immune cells to enter or exit the CNS. The findings of cytokines in this disorder support the involvement of the BBB. Here, we aim to review the function of NMDARs and the relationship between anti-NMDAR antibodies and anti-NMDAR encephalitis. We summarize the present knowledge of the composition of the BBB, especially by emphasizing the role of BBB components. Finally, we further provide a discussion on the impact of BBB dysfunction in anti-NMDAR encephalitis.

## 1 Introduction

Anti-NMDAR encephalitis is the most common antibody-related autoimmune disease, accounting for approximately 81% of autoimmune encephalitis cases (1). It was first described in 2007 by Dalmau (2). According to a survey, anti-NMDAR encephalitis is more common in young patients than any specific viral etiology (3). Most young and female patients are affected (4). This disease usually presents with acute behavioral changes, psychiatric symptoms, seizures, and memory deficits (5). The reduction in NMDAR surface density and synaptic localization by anti-NMDAR antibodies is thought to be the main mechanism of the disease (6). It also involves B cells in the CNS, which can produce antibodies intrathecally. In a normal state, a low level of circulating B cells passes across the BBB (7). However, in anti-NMDAR encephalitis, there is a significant increase in B cells. It is well known that the brain is devoid of autoimmune cells and antibodies due to the BBB. Impaired BBB may play an important role in this disease. Several CNS diseases involve BBB dysfunction, including neurodegenerative disorders (8), encephalitis (9), epilepsy (10), and multiple sclerosis (11). However, less attention has been given to the role of BBB in anti-NMDAR encephalitis.

In this review, we introduce the NMDA receptor and anti-NMDAR encephalitis. We then outline the structural and physiological roles of the BBB, including a set of BBB elements. The role of the BBB in therapy is also mentioned. Finally, we examine the role of the BBB in anti-NMDAR encephalitis. We hope to provide a novel perspective for understanding the pathogenesis of this disease.

## 2 Anti-NMDAR encephalitis and NMDA receptors

### 2.1 NMDA receptors

NMDAR, as a heterotetrametric transmembrane ion channel, is a subtype of glutamate receptor. It is formed by the combination of two necessary GluN1 subunits and two other subunits, including GluN2 or GluN3 subunits. It is widely expressed in various CNS cells (12), including neurons, endothelial cells, microglia, oligodendrocytes, and astrocytes. Here, we focus on its function in neurons and endothelial cells (the function of endothelial cells is described in part 3.1). In the CNS, NMDARs are predominantly expressed by neurons and are mainly distributed in the prefrontal cortex, hippocampus, amygdala, and hypothalamus (13). It widely exists in CNS neurons and is involved in a series of neural activities. As glutamate receptors, NMDARs can regulate the survival of neurons and the development of dendrites and axons. NMDARs are also involved in synaptic transmission and plasticity, as well as higher nervous activity such as learning, memory, and emotion (14, 15). However, excessive NMDAR activity leads to excitotoxicity and promotes cell death (16). Glutamate and glycine (or d-serine) are NMDAR agonists. NMDARs may also be blocked by magnesium. Activated NMDARs play an important role in regulating long-term potentiation (LTP) and long-term depression (LTD) of synaptic transmission (17) by mediating Ca^2+^ permeability.

It is believed that the functions of NMDARs mainly involve two kinds of diseases. Excitation is the first concern. It is associated with a series of diseases, such as stroke and epilepsy. Stimulation by glutamate of NMDARs can result in the death of neurons in these disorders. The other is NMDAR hypofunction, which may induce the elimination of synaptic activity mediated by NMDARs, resulting in memory, cognition, and behavioral defects. It is associated with schizophrenia and anti-NMDAR encephalitis (18, 19). The extracellular N-terminal domain of the NR1 subunit is the main targeting epitope (2). NMDAR hypofunction may induce a selective and reversible decrease in NMDAR quantity on the cell surface, mainly in the hippocampus (6, 19).

### 2.2 Anti-NMDAR encephalitis and anti-NMDAR antibodies

#### 2.2.1 Essential clinical characteristics of anti-NMDAR encephalitis

Anti-NMDAR encephalitis, characterized by CSF anti-NMDAR antibodies, is an antibody-related autoimmune encephalitis. Compared to other patients, anti-NMDAR encephalitis patients have a longer intensive care unit length of stay and a greater percentage of mental symptoms, motor disorders, and autonomic dysfunction (20). Following its initial reported in 2007, the number of diagnosed cases is increasing (2), and it is receiving increasing attention.

In general, anti-NMDAR encephalitis often affects young individuals and women. It also occurs in children and elderly individuals. This disease is commonly associated with ovarian teratoma and herpes simplex encephalitis. However, the mechanism is not very clear. The main symptoms of this disorder are mental symptoms, accompanied by memory impairment, autonomic nervous symptoms, seizures, and respiratory failure in over half of the patients. At the onset, psychiatric or behavioral symptoms are the most common, making it difficult to distinguish between anti-NMDAR encephalitis and primary psychosis (21). Sometimes, anti-NMDAR encephalitis is accompanied by other immune diseases, such as progressive systemic sclerosis (22) and myelin oligodendrocyte glycoprotein antibody-associated encephalitis (23).

Human autoimmunity plays an important role in this disorder. T lymphocytes and B lymphocytes have been found in brain samples from patients with anti-NMDAR encephalitis (24). Single-cell sequencing confirmed the activation and differentiation of B lymphocytes and the amplification of plasma cells in anti-NMDAR encephalitis (25). Mild perivascular lymphocytic cuffing, microglial activation, and a decrease in NMDAR density have been observed in the hippocampus during a brain biopsy or autopsy (2, 24). Compared to non-inflammatory neurological disease patients, the proportion of CD19+ B lymphocytes in the CSF of anti-NMDAR encephalitis patients is higher (26). Both oligoclonal bands and the production of many intrathecal immunoglobulins are characteristics of anti-NMDAR encephalitis (27–29). Obvious microglial proliferation and IgG deposition were detected in a series of brain regions, including the hippocampus and forebrain, accompanied by rare inflammatory infiltration (24, 30). Rituximab may relieve anti-NMDAR encephalitis in patients who fail to respond to first-line treatment (31). This provides evidence that B cells may be crucial for anti-NMDAR encephalitis development.

#### 2.2.2 Anti-NMDAR antibodies and their role in anti-NMDAR encephalitis

Antibodies against NMDAR play an important role in anti-NMDAR encephalitis. They are very common in the blood and CSF of patients with this disorder and are rarely present in other diseases. Studies have shown that anti-NMDAR antibodies from patients can selectively mediate the internalization of surface NMDARs, leading to a decrease in the surface density of synaptic NMDAR clusters and a reduction in glutamate synaptic function (6, 32). Initially, antibodies were detected in young women with teratomas. Ectopic neurons in teratomas are thought to be immune triggers and sources of autoantigens (2). Recent research shows that NMDAR antibodies may be produced from the tertiary lymphatic structure of tumors and traditional secondary lymphatic organs (33).

The anti-NMDAR antibody level is associated with the severity of disease symptoms, outcomes, and prognosis. Compared to patients without anti-NMDAR antibodies, patients with antibodies present with more aggression, exaltation, and disinhibition (20). Although there was no significant difference between severe patients and non-severe patients (*P* = 0.062), the strong positive rate of severe patients was higher than that of non-severe patients (48.7% vs. 29.2%) (34). There is also a certain correlation between antibody levels and symptoms. Patients with higher antibody titers were found to more commonly present a psychiatric symptom as the primary symptom and to display more severe clinical features than those with lower antibody titers. EEG background activity and symptoms were linked to CSF antibody titers. Serum titers decreased in patients with improved symptoms (19).

Currently, several anti-NMDAR encephalitis models are being built, including models induced by the herpes simplex encephalitis virus, selected peptides, patient CSF, and so on (35–37). All of these findings support the importance of antibodies in this disorder. Antibodies are so important for this disorder that they have been included in the anti-NMDAR encephalitis diagnostic criteria (38).

Although the important role of anti-NMDAR antibodies in this disease has been recognized, there is some controversy regarding the involvement of these antibodies in anti-NMDAR encephalitis. First, it has been proven that all natural anti-NMDAR1 antibodies have pathogenic potential (39, 40). The seroprevalence of anti-NMDAR antibodies was found to be similar between schizophrenic patients and healthy individuals (41). Second, the antibody titer is related to the severity, but alleviation of the disease may not be completely related to the decrease in titer. Studies have found that intrathecal synthesis can still exist for several years after symptoms recover (42). Although the CNS is an immune-privileged organ, peripheral diseases are known to affect the CNS. Some underlying tumors in the periphery may trigger several neurologic deficits, such as paraneoplastic neurological syndrome (43). The first anti-NMDAR encephalitis patient was diagnosed with ovarian teratoma in 2007 (2). The presence of nervous tissues in ovarian teratomas may play a role in the pathogenesis of anti-NMDAR encephalitis. The resection of teratomas is beneficial for the relief of symptoms in patients (44). The controversy surrounding the role of anti-NMDAR antibodies and the impact of the peripheral immune system on anti-NMDAR encephalitis is a topic that requires further investigation. Maybe, there is another mechanism that plays a role in this disease.

## 3 Structural, physiological, and pathological roles of the BBB

**Figure 1:**
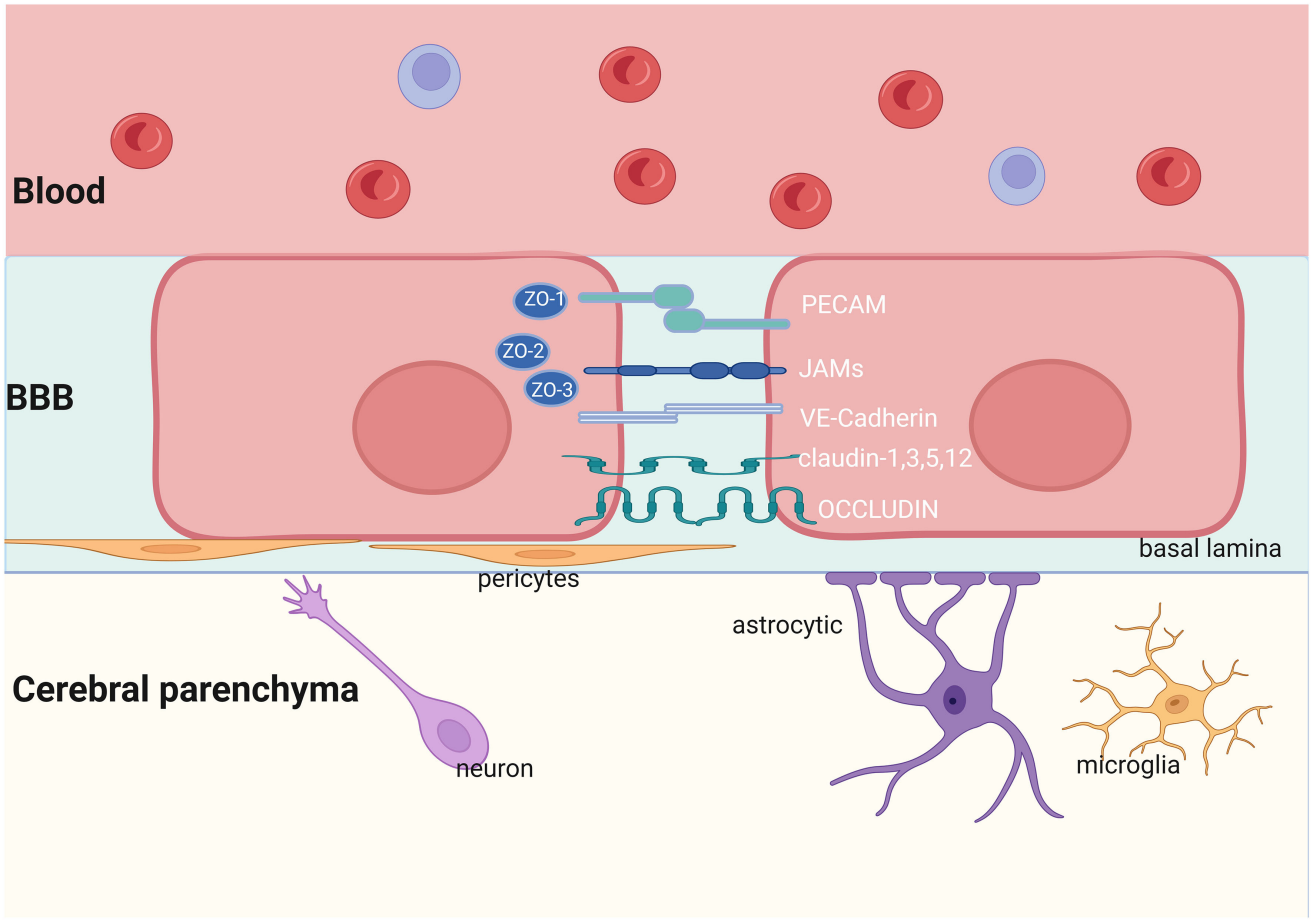
Structure of the BBB. The NVU is made up of neurons, astrocytes, microglia, pericytes, and endothelial cells. The TJs and AJs in the brain endothelial cells limit the transportation of proteins and other nutrients. JAMs, Junctional Adhesion Molecules; PECAM, Platelet Endothelial Cell Adhesion Molecule; VE-Cadherin, Vascular Endothelial Cadherin; ZO, Zonula Occludens. Created with BioRender.com.

The brain is separated from the circulatory system mainly by the BBB. It can regulate molecular transport between the CNS and blood, which is necessary to maintain homeostasis of the brain. The BBB is composed of brain microvascular endothelial cells, astrocytes, pericytes (PCs) and basement membrane (45) (Figure 1). Substances pass through the BBB in the following ways: (1) paracellular transport via damaged tight junction proteins (TJs) between endothelial cells and (2) transcellular transport via endocytosis vesicles or transport proteins (46). Neurons and microglia have the potential to influence the function of the BBB. Together with the elements of the BBB, they form the neurovascular unit (NVU), which plays a crucial role in maintaining the normal function of the CNS.

### 3.1 Endothelial cells

Endothelial cells (ECs) are the core components of the BBB and have always been a research hotspot for the BBB. Neurons, astrocytes, microglia, and pericytes influence BBB integrity by regulating the ECs. ECs lack endothelial fenestrations and have relatively few caveolar vesicles (47–49). They protect the brain by restricting paracellular and transcellular transport. Cytoplasmic proteins and transmembrane proteins, which are the focus of BBB research, determine the function and structural stability of TJs (50–53).

#### 3.1.1 Paracellular transport

TJs include claudin-5, occludin, and junction adhesion molecules, which promote the maintenance of cell polarity. Occludin, claudin-5, and ZO-1 are the main structural barrier proteins and are considered sensitive indicators of normal and disrupted BBB function (54).

##### 3.1.1.1 Claudin-5

Many elements of the claudin family with a molecular mass of 20–27 kDa are assumed to contribute to BBB integrity. Claudin-5 is the most enriched isoform in the claudin family (55). It is always expressed in ECs that form capillaries, which is the main components of TJs and is essential for maintaining BBB integrity (56, 57). Claudin-5 exclusively limits the paracellular permeability of molecules larger than the size-selective threshold (<800 Da) across the BBB (56). The *in vitro* and *in vivo* data indicate that the decrease in claudin-5 may cause BBB disruption (58). Claudin-5 knockout and redistribution from the membrane to the cytoplasm can lead to increased BBB permeability (56, 59). The expression and regulation of claudin-5 are influenced by multiple factors. Several factors, such as vascular endothelial (VE)-cadherin, glucocorticoids, glial cell line-derived neurotrophic factor (GDNF), and estrogen, can upregulate claudin-5 expression (60–63). Vascular endothelial growth factor (VEGF) (64) and transforming growth factor-b (TGF-b) (65) downregulate the expression of claudin-5, resulting in a decrease in BBB integrity. Autophagy may alleviate hypoxia-induced BBB injury by regulating claudin-5 redistribution in stroke (66). Other claudin elements, such as claudin-1,3 and claudin-11, are also associated with the BBB, although their levels are much lower than that of claudin-5 (67).

##### 3.1.1.2 Occludin

Occludin is the first confirmed TJ protein, with a molecular weight of 65 kDa (53). The expression level of occludin in ECs is higher than that in non-nerve tissues (68). Similar to claudin-5, it regulates the BBB through the paracellular pathway (53). However, it does not participate in the developmental regulation of the BBB and only maintains its integrity (69). Previous studies have shown that the downregulation of occludin can significantly reduce transcutaneous/transendothelial resistance (TEER) and increase paracellular diffusion (70). Compared to wild-type mice, the permeability of the BBB significantly increases in occludin-deficient mice after stroke (71). Many factors, such as matrix metalloproteinase-9 (MMP-9), ubiquitination, phosphorylation, tumor necrosis factor, IL-1β, and IFN-γ, participate in regulating the expression of occludin (69). Various signaling pathways are involved in the regulation of occludin, including the NF-κB, MAPK, PKC, RhoK, and ERK1/2 (72, 73). Vascular endothelial growth factor (VEGF) mediates MMP-9 activation to damage the BBB, leading to a decrease in the expression of occludin (74, 75). Autophagy also participates in BBB disruption by regulating occludin (70).

##### 3.1.1.3 ZO-1

Zonula occludens proteins (ZO-1, ZO-2, and ZO-3) share sequence similarities. ZO-1 and ZO-3 are cytoplasmic tight junctional accessory proteins that provide structural support to ECs (76) and are related to BBB dysfunction in many neurological diseases, such as stroke, subarachnoid hemorrhage, and Parkinson's disease (77–82). ZO-1 redistribution and reduction, mediated by autophagy and MMP-2/9, participate in BBB leakage in stroke. Endophilin-1 participates in regulating BBB permeability by controlling ZO-1 and occludin expression (83).

#### 3.1.2 Transcellular transport

In addition to paracellular transport, intercellular transport is another important way for substances to pass through the BBB. This approach is influenced by specific transport proteins, endocytosis, and diffusion movements in the endothelial cell membrane (84). Endocytosis includes internalization, sorting, and exocytosis. EC internalization may occur through vesicles, grid protein-dependent endocytosis, or grid protein-independent endocytosis pathways (85). Vesicles in brain endothelial cells (BECs) play an important role in the endocytosis of the BBB, although the number of vesicles in BECs is lower than that in peripheral endothelial cells (86). The vesicles contain the vesicle protein caveolin-1, which participates in the transcytosis of the BBB. The upregulation of caveolin-1 and caveolin-2 expression was found in BECs in the EAE model and stroke (87, 88).

### 3.2 Other cell elements of the BBB

Although ECs are thought to be the primary element of the BBB, they require contact with various NVU elements to establish BBB characteristics (89). Pericytes are blood vessel wall cells wrapped around the basement membrane of microvessels (see Figure 1). PCs make close contact with ECs through “peg-and-socket” junctions within a common basal lamina (90, 91). Pericytes can regulate BBB permeability by controlling the expression of TJs and adhesive junction proteins in the BBB and influencing vesicles (92). Mice with low pericyte coverage around endothelial cells have a disrupted BBB (93–95). PC dysfunction leads to a decrease in TJs, increasing BBB permeability, and a loss of basement membrane proteins (92, 93, 96). In response to inflammatory mediators, pericytes may secrete key molecules that influence the BBB. Pericyte-derived vitronectin is an extracellular matrix (ECM) protein. It regulates BBB integrity by binding to integrin a5 on endothelial cells to inhibit endocytosis (97). The downregulation of pericytes can lead to BBB dysfunction via transcellular transport, leading to leakage of a large amount of polymer substances, including IgG and albumin (90, 92).

The end feet of astrocytes ensheathe the microvasculature. Astrocytes may secrete several factors that have dual effects on maintaining BBB function. Various factors, including VEGF (98), MMPs, nitric oxide (NO), glutamate, and endothelin-1, may exacerbate the disruption of the BBB by regulating TJs. Other factors secreted by astrocytes may decrease BBB permeability, such as angiopoietin-1 (Ang-1), sonic hedgehog (Shh), GDNF, transforming growth factor-β (TGF-β), retinoic acid (RA), insulin-like growth factor-1, and apolipoprotein E (99, 100). Astrocyte-secreted Shhs have been shown to protect the BBB (101), which reinforce BBB junctional tightness by increasing TJ expression in ECs (102) and delaying BBB breakdown under pathological conditions. Induced by albumin extravasation, astrocytes also release MMP to degrade the basement membrane, resulting in BBB dysfunction (103).

Microglia are resident CNS macrophages (104). Microglia are widely distributed in the brain tissue, including the basal ganglia, hippocampus, substantia nigra, and olfactory brain (105). They exist at vascular junctions and bridge endothelial tip cells, monitoring BBB integrity and the inflow of agents into the brain (106). It can synthesize many proinflammatory cytokines, directly affecting the permeability of the BBB (107). Microglia also have a dual effect on the BBB. M1-type microglia may damage BBB function, while M2 anti-inflammatory microglia play a protective role in the BBB (108, 109).

ECs may lose their BBB properties when cultured alone. They may show enhanced TEER and begin to express many TJs in coculture with astrocytes and pericytes (109). Endothelial cells cocultured with resting microglia or astrocytes express more occludin and ZO-1 (85). Endothelial TJs are also tighter in the presence of pericytes. A recent study demonstrated that PCs may transfer signals to ECs through ligand–receptor interactions, which is an important mechanism for regulating BBB permeability (97). Astrocytic end feet are important for the establishment and maintenance of the BBB (91). They play an important role in preventing excessive immune cells from entering the space around blood vessels.

### 3.3 BBB and disease

The BBB exists widely in the brain and is involved in CNS diseases, including epilepsy, cerebrovascular accidents, and mental disorders. It provides immune preservation of the CNS. Any damage to the BBB will result in adverse consequences, leading to diseases, or aggravation of illness. The role of the BBB in stroke has been widely researched. A large amount of evidence suggests that inflammation after ischemia is associated with BBB disruption, vascular edema, hemorrhagic transformation, and a poorer neurological prognosis. Occludin is mediated by MMPs (110), nitric oxide synthase (111), reactive oxygen species (112), and rho kinase (113). VEGF may affect the permeability of the BBB after cerebral ischemia and reperfusion. BBB dysfunction is closely associated with the onset of Alzheimer's disease (AD). Glucose transporter1 (GLUT1) in the ECs of AD patients is lower than in the control group (114). The deficiency of GLUT1 in AD mice may lead to BBB dysfunction and a decrease in TJ protein (115). Multiple sclerosis (MS) is a CNS disease associated with immunity. BBB destruction and immune cell infiltration into the CNS are characteristics of MS. Reduced or interrupted staining of occludin, claudin-5, and VE cadherin has been observed in the brain tissues of MS patients on autopsy (116–118). Brain microvascular endothelial cell-like cells derived from MS showed increased BBB permeability and decreased TJ integrity (119). Immune cell migration across the endothelial barrier is generally mediated by the coordinated binding of adhesion molecules and the interaction of chemokines/chemokine receptors, as well as the action of MMPs and their tissue inhibitors (TIMPs) (120). ECs alter their immune phenotype by upregulating intercellular adhesion molecule-1 (ICAM-1), vascular cell adhesion molecule-1 (VCAM-1), and atypical chemokine receptor 1 (ACKR1), allowing more immune cells to infiltrate the CNS (121–124). T cells infiltrate the CNS mainly by transcellular transport (125). B-cell migration across ECs is faster than that of T cells. It may be regulated by the adhesion molecules VLA-4 and ICAM-1, the chemokines monocyte chemoattractant protein-1 and IL-8, and TIMP-1 (126).

The BBB exists widely in the brain and is involved in neural microenvironmental homeostasis. It provides immune preservation of the CNS. On the other hand, it is the main obstacle to the treatment of most CNS diseases (127). Only a few CNS disorders may be treated by small-molecule drugs, which cross the BBB (128). The human immunodeficiency virus in the periphery may be significantly reduced by antiretroviral therapy (129). However, owing to the presence of the BBB, antiretroviral drugs cannot be used in the brain (130). How to overcome the BBB is a challenge in the therapeutic development of CNS diseases (131). New strategies and medicines are currently being developed to resolve this problem.

## 4 BBB, NMDARs, and anti-NMDAR encephalitis

### 4.1 NMDARs and BBB

NMDARs may be activated to induce a breakdown of the BBB (132). Overactivation of NMDARs can alter the expression of TJs, affecting BBB permeability (133). Circulating tPA can activate endothelial NMDARs and increase BBB permeability via the Rho signaling pathway (133, 134). In addition, the activation of NMDARs can disrupt the BBB by activating the MEK1/2-ERK1/2 signaling pathway and upregulating MMP2/9 expression (135, 136).

In addition to affecting paracellular pathways, NMDARs can also affect the BBB via transcellular transport. Treatment with NMDA can increase intercellular movement. Many bioactive molecules, including transferrin, glucose, and immunoglobulin, enter the brain through the BBB. It may be achieved via clathrin and caveolin, which are inhibited by anti-NMDAR antibodies. NMDAR-deficient ECs result in decreased neuronal density and brain vasculature (137).

There is evidence indicating the existence of NMDAR in astrocytes (138). The expression of NMDARs in astrocytes may be upregulated in transient ischemic astrocytes (139). Activated astrocytic NMDARs may mediate ion currents and intracellular Ca^2+^ waves (140) and contribute to glial postsynaptic currents (141). Some studies have shown that activated NMDARs in astrocytes also stimulate the secretion of proinflammatory cytokines (142, 143).

The expression of NMDARs was also observed in oligodendrocytes and microglia. These cells are involved in the development of myelin, the regulation of glucose transporters, and glucose trafficking (144). NMDAR currents in oligodendrocytes exist in multiple brain regions and at various developmental stages, which are involved in excitotoxicity mechanisms (145, 146). The role of glucose transporters has been confirmed in anti-NMDAR encephalitis (147). Excessive activation of oligodendrocyte NMDARs may trigger excitotoxic cell death via Ca^2+^ overload and energy metabolism dysfunction (148). Microglial NMDAR may combine with NMDA, inducing microglial activation in hypertrophic and amoeboid states and the release of proinflammatory factors.

### 4.2 The BBB in anti-NMDAR encephalitis

**Figure 2:**
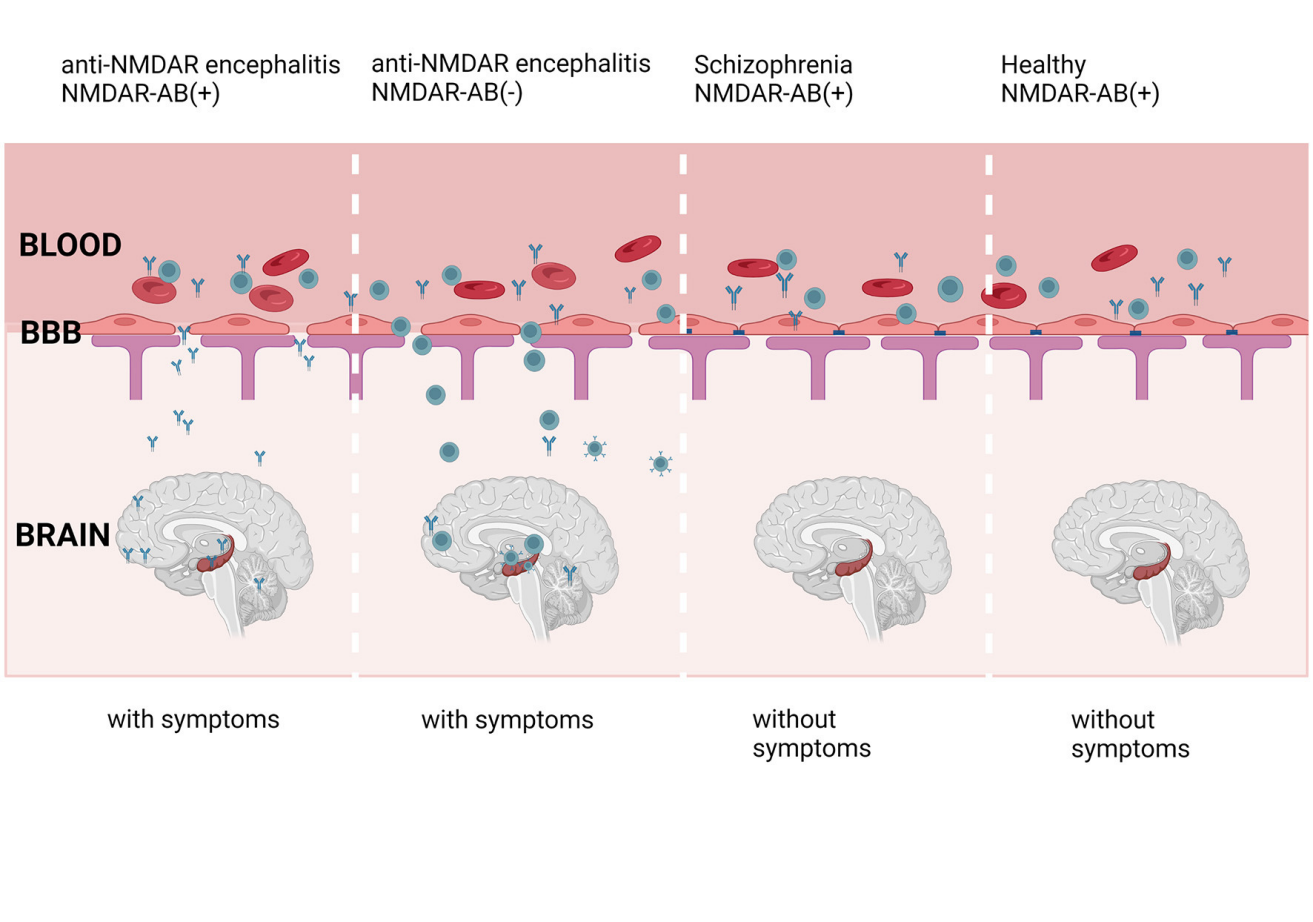
NMDAR-ab in the blood is discovered in anti-NMDAR encephalitis patients, healthy individuals, and patients with schizophrenia. However, the symptoms observed in those patients and healthy individuals are not similar to anti-NMDAR encephalitis. The symptoms may be caused by CSF antibodies, which come from blood. The impaired BBB allows the antibodies and B cells in serum to enter the brain, leading to the appearance of symptoms. Created with BioRender.com.

The role of antibodies in anti-NMDAR encephalitis is well known. However, whether CNS NMDARs or circulating NMDARs participate in the disease is unknown. Several studies reported that the seropositive prevalence of anti-NMDAR1 antibodies is similar in both healthy individuals and psychotic patients (149, 150). All naturally occurring NMDAR1 antibodies have pathogenic potential (39). However, not all patients and healthy individuals with NMDAR antibodies show symptoms. The antibodies cause behavioral phenotypes only when they enter the CNS (see Figure 2). The antibody in CSF was more important than the serum-derived antibody. The titer change in CSF was more closely related to relapses, outcomes, and patient condition than in serum (151, 152).

The antibody in CSF was more important than the serum-derived antibody. Where does the anti-NMDAR antibody in the CNS come from? Antibodies in serum may enter the brain through the damaged BBB (see Figure 2) (39, 41, 150). Clonally expanded plasma cells in the CNS have been suggested as another source of CSF NMDAR antibodies (153). Interestingly, these B cells are reported to originate from peripheral lymphoid organs (33, 37). BBB impairment is important for the entry of autoimmune cells into the brain. As a barrier to isolating brain tissue and the peripheral circulatory system, the BBB plays a significant role.

The clinical symptoms of anti-NMDAR encephalitis indicate that BBB impairment is a crucial step in the onset of the disease. The CSF albumin/serum albumin (QAlb) is one of the indicators of BBB damage. Yu et al. observed BBB dysfunction based on the results of QAlb in anti-NMDAR encephalitis (154). Some chemical agents related to BBB damage and CNS inflammation are observed in the CSF and serum in anti-NMDAR encephalitis (155, 156). Due to the impaired BBB, anti-NMDAR antibodies may transfer from pregnant C57BL/6J mice to fetuses, causing severe but reversible synaptic and neurodevelopmental alterations (157). A study showed that compared to wild-type mice, apoE-/- mice with a disrupted BBB respond to intravenous NMDAR antibodies, showing symptoms (150, 158). It was confirmed that BBB damage was present in anti-NMDAR encephalitis mice (159).

The integrity of the BBB is related not only to the occurrence of anti-NMDAR encephalitis but also to the prognosis and severity of symptoms. According to previous reports, BBB dysfunction is associated with prognosis and mRS scores after 2 months of follow-up (154). The symptoms of anti-NMDAR encephalitis are associated with BBB dysfunction (41). Gong et al. reported that PI3K inhibition can improve neurological behavior by alleviating BBB and neuron injury (159). Interestingly, circulating antibodies, passing through the intact BBB at a low rate and titer, cannot cause obvious symptoms. The antibodies may combine tightly with the brain tissue, preventing discharge into the CSF (160). However, many antibodies enter the brain through a damaged BBB, causing clinical symptoms in anti-NMDAR encephalitis.

Although the BBB plays an important role in anti-NMDAR encephalitis, the mechanism is unclear. There are also other antibodies present in the cerebrospinal fluid of patients with anti-NMDAR encephalitis (161). These antibodies can react with different types of blood vessels. *In vitro* and *in vivo* experiments have confirmed the presence of the vascular targeting antibody mAb011-138 in the CSF of patients with NMDAR encephalitis. It can react with small blood vessels and increase BBB permeability by downregulating occludin (162).

## 5 Conclusion

Although anti-NMDAR encephalitis is a rare disorder, it causes a series of serious symptoms and is easily misdiagnosed as psychosis. It often affects young individuals and has attracted increasing attention. Anti-NMDAR antibodies, especially those in the CSF, play a key role in this disorder. The presence and concentration of antibodies in CSF may influence the onset and severity of this disorder. The BBB is an important physical and metabolic barrier that controls the entry of agents into the brain. BBB dysfunction is not only associated with the entry of serum antibodies and immune cells but is also linked to the symptoms caused by antibodies. BBB breakdown has been thought to be an important event in anti-NMDAR encephalitis. However, the mechanism is unclear, and only a few studies on BBB disruption in anti-NMDAR encephalitis have been reported. The association between the elements of the BBB and NMDARs, including astrocytes, microglia, pericytes, and endothelial cells, is still unclear. The BBB has also been linked to the treatment of many CNS diseases. NMDARs are involved in BBB dysfunction in other disorders. It is still unknown whether anti-NMDAR antibodies affect the BBB in anti-NMDAR encephalitis. The repair of the BBB or the production of new medicines across the BBB for anti-NMDAR encephalitis is also important. Future studies investigating anti-NMDAR encephalitis should consider the factors that affect BBB integrity.

## Author contributions

XG: Writing—original draft. NW: Writing—review & editing. HZ: Writing—review & editing. NT: Writing—review & editing. KW: Writing—review & editing. QM: Writing—review & editing.

## References

[1] Gu Y Zhong M He L Li W Huang Y Liu J. Epidemiology of antibody-positive autoimmune encephalitis in southwest china: a multicenter study. Front Immunol. (2019) 10:2611. 10.3389/fimmu.2019.02611 31781111 PMC6861323

[2] Dalmau J Tuzun E Wu HY Masjuan J Rossi JE Voloschin A. Paraneoplastic anti-N-methyl-D-aspartate receptor encephalitis associated with ovarian teratoma. Ann Neurol. (2007) 61:25–36. 10.1002/ana.21050 17262855 PMC2430743

[3] Gable MS Sheriff H Dalmau J Tilley DH Glaser CA. The frequency of autoimmune N-methyl-D-aspartate receptor encephalitis surpasses that of individual viral etiologies in young individuals enrolled in the California Encephalitis Project. Clin Infect Dis. (2012) 54:899–904. 10.1093/cid/cir1038 22281844 PMC3297648

[4] Dalmau J Armangue T Planaguma J Radosevic M Mannara F Leypoldt F. An update on anti-NMDA receptor encephalitis for neurologists and psychiatrists: mechanisms and models. Lancet Neurol. (2019) 18:1045–57. 10.1016/S1474-4422(19)30244-3 31326280

[5] Wandinger K Saschenbrecker S Stoecker W Dalmau J. Anti-NMDA-receptor encephalitis: a severe, multistage, treatable disorder presenting with psychosis. J Neuroimmunol. (2011) 231:86–91. 10.1016/j.jneuroim.2010.09.012 20951441

[6] Hughes EG Peng X Gleichman AJ Lai M Zhou L Tsou R. Cellular and synaptic mechanisms of anti-NMDA receptor encephalitis. J Neurosci. (2010) 30:5866–75. 10.1523/JNEUROSCI.0167-10.2010 20427647 PMC2868315

[7] Alter A Duddy M Hebert S Biernacki K Prat A Antel JP. Determinants of human B cell migration across brain endothelial cells. J Immunol. (2003) 170:4497–505. 10.4049/jimmunol.170.9.4497 12707326

[8] Sweeney MD Sagare AP Zlokovic BV. Blood-brain barrier breakdown in Alzheimer disease and other neurodegenerative disorders. Nat Rev Neurol. (2018) 14:133–50. 10.1038/nrneurol.2017.188 29377008 PMC5829048

[9] Liu H Qiu K He Q Lei Q Lu W. Mechanisms of blood-brain barrier disruption in herpes simplex encephalitis. J Neuroimmune Pharmacol. (2019) 14:157–72. 10.1007/s11481-018-9821-6 30456443

[10] Dadas A Janigro D. Breakdown of blood brain barrier as a mechanism of post-traumatic epilepsy. Neurobiol Dis. (2019) 123:20–6. 10.1016/j.nbd.2018.06.022 30030025 PMC6794150

[11] Balasa R Barcutean L Mosora O Manu D. Reviewing the significance of blood-brain barrier disruption in multiple sclerosis pathology and treatment. Int J Mol Sci. (2021) 22:8370. 10.3390/ijms22168370 34445097 PMC8395058

[12] Hogan-Cann AD Anderson CM. Physiological roles of non-neuronal NMDA receptors. Trends Pharmacol Sci. (2016) 37:750–67. 10.1016/j.tips.2016.05.012 27338838

[13] Bersier MG Pena C Rodriguez DLAG. The expression of NMDA receptor subunits in cerebral cortex and hippocampus is differentially increased by administration of endobain E, a Na+, K+-ATPase inhibitor. Neurochem Res. (2008) 33:66–72. 10.1007/s11064-007-9412-z 17680361

[14] Fouad IA Sharaf NM Abdelghany RM El SN. Neuromodulatory effect of thymoquinone in attenuating glutamate-mediated neurotoxicity targeting the amyloidogenic and apoptotic pathways. Front Neurol. (2018) 9:236. 10.3389/fneur.2018.00236 29706929 PMC5908889

[15] Montes DOBP. Flux-independent NMDAR signaling: molecular mediators, cellular functions, and complexities. Int J Mol Sci. (2018) 19:3800. 10.3390/ijms19123800 30501045 PMC6321296

[16] Wang R Reddy PH. Role of glutamate and NMDA receptors in Alzheimer's disease. J Alzheimers Dis. (2017) 57:1041–8. 10.3233/JAD-160763 27662322 PMC5791143

[17] Bliss TV Collingridge GL. A synaptic model of memory: long-term potentiation in the hippocampus. Nature. (1993) 361:31–9. 10.1038/361031a0 8421494

[18] Balu DT Coyle JT. The NMDA receptor 'glycine modulatory site' in schizophrenia: D-serine, glycine, and beyond. Curr Opin Pharmacol. (2015) 20:109–15. 10.1016/j.coph.2014.12.004 25540902 PMC4805108

[19] Dalmau J Gleichman AJ Hughes EG Rossi JE Peng X Lai M. Anti-NMDA-receptor encephalitis: case series and analysis of the effects of antibodies. Lancet Neurol. (2008) 7:1091–8. 10.1016/S1474-4422(08)70224-2 18851928 PMC2607118

[20] Chen X Li JM Liu F Wang Q Zhou D Lai X. Anti-N-methyl-D-aspartate receptor encephalitis: a common cause of encephalitis in the intensive care unit. Neurol Sci. (2016) 37:1993–8. 10.1007/s10072-016-2702-y 27620725

[21] Kayser MS Titulaer MJ Gresa-Arribas N Dalmau J. Frequency and characteristics of isolated psychiatric episodes in anti-N-methyl-d-aspartate receptor encephalitis. JAMA Neurol. (2013) 70:1133–9. 10.1001/jamaneurol.2013.3216 23877059 PMC3809325

[22] Suzuki H Samukawa M Kitada M Ichihashi J Mistui Y Tanaka K. A case of anti-N-methyl-D-aspartate receptor encephalitis with systemic sclerosis. Eur J Neurol. (2011) 18:e145–6. 10.1111/j.1468-1331.2011.03485.x 21985028

[23] Wang M Tan J Zhou Z Wang Y Bako SY Yang Y. Relapsing MOG-IgG-associated diseases coexisting with anti-NMDAR encephalitis: a case report and literature review. J Integr Neurosci. (2022) 21:82. 10.31083/j.jin2103082 35633163

[24] Camdessanché JP Streichenberger N Cavillon G Rogemond V Jousserand G Honnorat J. Brain immunohistopathological study in a patient with anti-NMDAR encephalitis. Eur J Neurol. (2011) 18:929–31. 10.1111/j.1468-1331.2010.03180.x 20722705

[25] Jiang Y Dai S Jia L Qin L Zhang M Liu H. Single-cell transcriptomics reveals cell type-specific immune regulation associated with anti-NMDA receptor encephalitis in humans. Front Immunol. (2022) 13:1075675. 10.3389/fimmu.2022.1075675 36544777 PMC9762154

[26] Dale RC Pillai S Brilot F. Cerebrospinal fluid CD19(+) B-cell expansion in N-methyl-D-aspartate receptor encephalitis. Dev Med Child Neurol. (2013) 55:191–3. 10.1111/dmcn.12036 23151040

[27] Malter MP Elger CE Surges R. Diagnostic value of CSF findings in antibody-associated limbic and anti-NMDAR-encephalitis. Seizure. (2013) 22:136–40. 10.1016/j.seizure.2012.12.013 23318046

[28] Sudan YS Vinayan KP Roy AG Wagh A Kannoth S Patil S. Clinical characteristics and follow-up of south indian children with autoimmune encephalopathy. Indian J Pediatr. (2016) 83:1367–73. 10.1007/s12098-016-2092-4 27086607

[29] Durr M Nissen G Suhs KW Schwenkenbecher P Geis C Ringelstein M. CSF findings in acute NMDAR and LGI1 antibody-associated autoimmune encephalitis. Neurol Neuroimmunol Neuroinflamm. (2021) 8:6. 10.1212/NXI.0000000000001086 34697224 PMC8546742

[30] Tuzun E Zhou L Baehring JM Bannykh S Rosenfeld MR Dalmau J. Evidence for antibody-mediated pathogenesis in anti-NMDAR encephalitis associated with ovarian teratoma. Acta Neuropathol. (2009) 118:737–43. 10.1007/s00401-009-0582-4 19680671 PMC2888642

[31] Dhawan SR Sankhyan N. Low-dose rituximab in children with anti-NMDAR encephalitis. Pediatr Neurol. (2018) 87:82. 10.1016/j.pediatrneurol.2018.07.006 30501890

[32] Mikasova L De Rossi P Bouchet D Georges F Rogemond V Didelot A. Disrupted surface cross-talk between NMDA and Ephrin-B2 receptors in anti-NMDA encephalitis. Brain. (2012) 135:1606–21. 10.1093/brain/aws092 22544902

[33] Al-Diwani A Theorell J Damato V Bull J McGlashan N Green E. Cervical lymph nodes and ovarian teratomas as germinal centres in NMDA receptor-antibody encephalitis. Brain. (2022) 145:2742–54. 10.1093/brain/awac088 35680425 PMC9486890

[34] Zhang Y Liu G Jiang M Chen W He Y Su Y. Clinical characteristics and prognosis of severe anti-N-methyl-D-aspartate receptor encephalitis patients. Neurocrit Care. (2018) 29:264–72. 10.1007/s12028-018-0536-6 29651625

[35] Malviya M Barman S Golombeck KS Planaguma J Mannara F Strutz-Seebohm N. NMDAR encephalitis: passive transfer from man to mouse by a recombinant antibody. Ann Clin Transl Neurol. (2017) 4:768–83. 10.1002/acn3.444 29159189 PMC5682115

[36] Linnoila J Pulli B Armangue T Planaguma J Narsimhan R Schob S. Mouse model of anti-NMDA receptor post-herpes simplex encephalitis. Neurol Neuroimmunol Neuroinflamm. (2019) 6:e529. 10.1212/NXI.0000000000000529 30697582 PMC6340334

[37] Wagnon I Helie P Bardou I Regnauld C Lesec L Leprince J. Autoimmune encephalitis mediated by B-cell response against N-methyl-d-aspartate receptor. Brain. (2020) 143:2957–72. 10.1093/brain/awaa250 32893288

[38] Graus F Titulaer MJ Balu R Benseler S Bien CG Cellucci T. A clinical approach to diagnosis of autoimmune encephalitis. Lancet Neurol. (2016) 15:391–404. 10.1016/S1474-4422(15)00401-9 26906964 PMC5066574

[39] Castillo-Gomez E Oliveira B Tapken D Bertrand S Klein-Schmidt C Pan H. All naturally occurring autoantibodies against the NMDA receptor subunit NR1 have pathogenic potential irrespective of epitope and immunoglobulin class. Mol Psychiatry. (2017) 22:1776–84. 10.1038/mp.2016.125 27502473

[40] Ehrenreich H. Autoantibodies against the N-Methyl-d-aspartate receptor subunit NR1: untangling apparent inconsistencies for clinical practice. Front Immunol. (2017) 8:181. 10.3389/fimmu.2017.00181 28298911 PMC5331041

[41] Hammer C Stepniak B Schneider A Papiol S Tantra M Begemann M. Neuropsychiatric disease relevance of circulating anti-NMDA receptor autoantibodies depends on blood-brain barrier integrity. Mol Psychiatry. (2014) 19:1143–9. 10.1038/mp.2013.110 23999527

[42] Lynch DR Rattelle A Dong YN Roslin K Gleichman AJ Panzer JA. Anti-NMDA Receptor encephalitis: clinical features and basic mechanisms. Adv Pharmacol. (2018) 82:235–60. 10.1016/bs.apha.2017.08.005 29413523

[43] Zaborowski MP Spaczynski M Nowak-Markwitz E Michalak S. Paraneoplastic neurological syndromes associated with ovarian tumors. J Cancer Res Clin Oncol. (2015) 141:99–108. 10.1007/s00432-014-1745-9 24965744 PMC4282879

[44] Liu Y Tian Y Guo R Xu X Zhang M Li Z. Anti-NMDA receptor encephalitis: retrospective analysis of 15 cases, literature review, and implications for gynecologists. J Healthc Eng. (2022) 2022:4299791. 10.1155/2022/4299791 35340259 PMC8941556

[45] Uemura MT Maki T Ihara M Lee V Trojanowski JQ. Brain microvascular pericytes in vascular cognitive impairment and dementia. Front Aging Neurosci. (2020) 12:80. 10.3389/fnagi.2020.00080 32317958 PMC7171590

[46] Zhao Z Nelson AR Betsholtz C Zlokovic BV. Establishment and dysfunction of the blood-brain barrier. Cell. (2015) 163:1064–78. 10.1016/j.cell.2015.10.067 26590417 PMC4655822

[47] Sedlakova R Shivers RR Del MR. Ultrastructure of the blood-brain barrier in the rabbit. J Submicrosc Cytol Pathol. (1999) 31:149–61. 10363362

[48] Kniesel U Wolburg H. Tight junctions of the blood-brain barrier. Cell Mol Neurobiol. (2000) 20:57–76. 10.1023/A:1006995910836 10690502 PMC11537529

[49] Abbott NJ Patabendige AA Dolman DE Yusof SR Begley DJ. Structure and function of the blood-brain barrier. Neurobiol Dis. (2010) 37:13–25. 10.1016/j.nbd.2009.07.030 19664713

[50] Hawkins BT Davis TP. The blood-brain barrier/neurovascular unit in health and disease. Pharmacol Rev. (2005) 57:173–85. 10.1124/pr.57.2.4 15914466

[51] Feldman GJ Mullin JM Ryan MP. Occludin: structure, function and regulation. Adv Drug Deliv Rev. (2005) 57:883–917. 10.1016/j.addr.2005.01.009 15820558

[52] Bauer H Zweimueller-Mayer J Steinbacher P Lametschwandtner A Bauer HC. The dual role of zonula occludens (ZO) proteins. J Biomed Biotechnol. (2010) 2010:402593. 10.1155/2010/402593 20224657 PMC2836178

[53] Furuse M Hirase T Itoh M Nagafuchi A Yonemura S Tsukita S. Occludin: a novel integral membrane protein localizing at tight junctions. J Cell Biol. (1993) 123:1777–88. 10.1083/jcb.123.6.1777 8276896 PMC2290891

[54] Willis CL Leach L Clarke GJ Nolan CC Ray DE. Reversible disruption of tight junction complexes in the rat blood-brain barrier, following transitory focal astrocyte loss. Glia. (2004) 48:1–13. 10.1002/glia.20049 15326610

[55] Ohtsuki S Sato S Yamaguchi H Kamoi M Asashima T Terasaki T. Exogenous expression of claudin-5 induces barrier properties in cultured rat brain capillary endothelial cells. J Cell Physiol. (2007) 210:81–6. 10.1002/jcp.20823 16998798

[56] Nitta T Hata M Gotoh S Seo Y Sasaki H Hashimoto N. Size-selective loosening of the blood-brain barrier in claudin-5-deficient mice. J Cell Biol. (2003) 161:653–60. 10.1083/jcb.200302070 12743111 PMC2172943

[57] Ahn JC Hwang SJ Lee HJ Kim KW. Claudin-5a knockdown attenuates blood-neural barrier in zebrafish. Comp Biochem Physiol C Toxicol Pharmacol. (2021) 250:109176. 10.1016/j.cbpc.2021.109176 34500089

[58] Koto T Takubo K Ishida S Shinoda H Inoue M Tsubota K. Hypoxia disrupts the barrier function of neural blood vessels through changes in the expression of claudin-5 in endothelial cells. Am J Pathol. (2007) 170:1389–97. 10.2353/ajpath.2007.060693 17392177 PMC1829471

[59] Liao Z Yang Z Piontek A Eichner M Krause G Li L. Specific binding of a mutated fragment of Clostridium perfringens enterotoxin to endothelial claudin-5 and its modulation of cerebral vascular permeability. Neuroscience. (2016) 327:53–63. 10.1016/j.neuroscience.2016.04.013 27095710

[60] Gavard J Gutkind JS. VE-cadherin and claudin-5: it takes two to tango. Nat Cell Biol. (2008) 10:883–5. 10.1038/ncb0808-883 18670447 PMC2666287

[61] Na W Shin JY Lee JY Jeong S Kim WS Yune TY. Dexamethasone suppresses JMJD3 gene activation via a putative negative glucocorticoid response element and maintains integrity of tight junctions in brain microvascular endothelial cells. J Cereb Blood Flow Metab. (2017) 37:3695–708. 10.1177/0271678X17701156 28338398 PMC5718327

[62] Shimizu F Sano Y Saito K Abe MA Maeda T Haruki H. Pericyte-derived glial cell line-derived neurotrophic factor increase the expression of claudin-5 in the blood-brain barrier and the blood-nerve barrier. Neurochem Res. (2012) 37:401–9. 10.1007/s11064-011-0626-8 22002662

[63] Shin JA Yoon JC Kim M Park EM. Activation of classical estrogen receptor subtypes reduces tight junction disruption of brain endothelial cells under ischemia/reperfusion injury. Free Radic Biol Med. (2016) 92:78–89. 10.1016/j.freeradbiomed.2016.01.010 26784014

[64] Shimizu F Sano Y Tominaga O Maeda T Abe MA Kanda T. Advanced glycation end-products disrupt the blood-brain barrier by stimulating the release of transforming growth factor-beta by pericytes and vascular endothelial growth factor and matrix metalloproteinase-2 by endothelial cells *in vitro*. Neurobiol Aging. (2013) 34:1902–12. 10.1016/j.neurobiolaging.2013.01.012 23428182

[65] Watabe T Nishihara A Mishima K Yamashita J Shimizu K Miyazawa K. TGF-beta receptor kinase inhibitor enhances growth and integrity of embryonic stem cell-derived endothelial cells. J CELL BIOL. (2003) 163:1303–11. 10.1083/jcb.200305147 14676305 PMC2173713

[66] Yang Z Lin P Chen B Zhang X Xiao W Wu S. Autophagy alleviates hypoxia-induced blood-brain barrier injury via regulation of CLDN5 (claudin 5). Autophagy. (2021) 17:3048–67. 10.1080/15548627.2020.1851897 33280500 PMC8526012

[67] Krause G Winkler L Mueller SL Haseloff RF Piontek J Blasig IE. Structure and function of claudins. Biochim Biophys Acta. (2008) 1778:631–45. 10.1016/j.bbamem.2007.10.018 18036336

[68] Hirase T Staddon JM Saitou M Ando-Akatsuka Y Itoh M Furuse M. Occludin as a possible determinant of tight junction permeability in endothelial cells. J Cell Sci. (1997) 110:1603–13. 10.1242/jcs.110.14.1603 9247194

[69] Yuan S Liu KJ Qi Z. Occludin regulation of blood-brain barrier and potential therapeutic target in ischemic stroke. Brain Circ. (2020) 6:152–62. 10.4103/bc.bc_29_20 33210038 PMC7646391

[70] Kim KA Kim D Kim JH Shin YJ Kim ES Akram M. Autophagy-mediated occludin degradation contributes to blood-brain barrier disruption during ischemia in bEnd3 brain endothelial cells and rat ischemic stroke models. Fluids Barriers CNS. (2020) 17:21. 10.1186/s12987-020-00182-8 32169114 PMC7071658

[71] Sugiyama S Sasaki T Tanaka H Yan H Ikegami T Kanki H. The tight junction protein occludin modulates blood-brain barrier integrity and neurological function after ischemic stroke in mice. Sci Rep. (2023) 13:2892. 10.1038/s41598-023-29894-1 36806348 PMC9938878

[72] Cohen D Dodds R Viberti G. Effect of protein restriction in insulin dependent diabetics at risk of nephropathy. Br Med J (Clin Res Ed). (1987) 294:795–8. 10.1136/bmj.294.6575.795 PMC1245861 3105747

[73] Kimura K Teranishi S Nishida T. Interleukin-1beta-induced disruption of barrier function in cultured human corneal epithelial cells. Invest Ophthalmol Vis Sci. (2009) 50:597–603. 10.1167/iovs.08-2606 19171646

[74] Bauer AT Burgers HF Rabie T Marti HH. Matrix metalloproteinase-9 mediates hypoxia-induced vascular leakage in the brain via tight junction rearrangement. J Cereb Blood Flow Metab. (2010) 30:837–48. 10.1038/jcbfm.2009.248 19997118 PMC2949161

[75] Zhao H Zheng T Yang X Fan M Zhu L Liu S. Cryptotanshinone attenuates oxygen-glucose deprivation/recovery-induced injury in an *in vitro* model of neurovascular unit. Front Neurol. (2019) 10:381. 10.3389/fneur.2019.00381 31057477 PMC6482155

[76] Haskins J Gu L Wittchen ES Hibbard J Stevenson BR. ZO-3, a novel member of the MAGUK protein family found at the tight junction, interacts with ZO-1 and occludin. J Cell Biol. (1998) 141:199–208. 10.1083/jcb.141.1.199 9531559 PMC2132714

[77] Katsuno T Umeda K Matsui T Hata M Tamura A Itoh M. Deficiency of zonula occludens-1 causes embryonic lethal phenotype associated with defected yolk sac angiogenesis and apoptosis of embryonic cells. Mol Biol Cell. (2008) 19:2465–75. 10.1091/mbc.e07-12-1215 18353970 PMC2397322

[78] Kaya M Palanduz A Kalayci R Kemikler G Simsek G Bilgic B. Effects of lipopolysaccharide on the radiation-induced changes in the blood-brain barrier and the astrocytes. Brain Res. (2004) 1019:105–12. 10.1016/j.brainres.2004.05.102 15306244

[79] Arican N Kaya M Kalayci R Uzun H Ahishali B Bilgic B. Effects of lipopolysaccharide on blood-brain barrier permeability during pentylenetetrazole-induced epileptic seizures in rats. Life Sci. (2006) 79:1–7. 10.1016/j.lfs.2005.12.035 16434059

[80] Fujii M Duris K Altay O Soejima Y Sherchan P Zhang JH. Inhibition of Rho kinase by hydroxyfasudil attenuates brain edema after subarachnoid hemorrhage in rats. Neurochem Int. (2012) 60:327–33. 10.1016/j.neuint.2011.12.014 22226843 PMC3288616

[81] Liu J Wang F Liu S Du J Hu X Xiong J. Sodium butyrate exerts protective effect against Parkinson's disease in mice via stimulation of glucagon like peptide-1. J Neurol Sci. (2017) 381:176–81. 10.1016/j.jns.2017.08.3235 28991675

[82] Tanaka K Matsumoto S Yamada T Yamasaki R Suzuki M Kido MA. Reduced Post-ischemic Brain Injury in Transient Receptor Potential Vanilloid 4 Knockout Mice. Front Neurosci. (2020) 14:453. 10.3389/fnins.2020.00453 32477057 PMC7235376

[83] Liu W Wang P Shang C Chen L Cai H Ma J. Endophilin-1 regulates blood-brain barrier permeability by controlling ZO-1 and occludin expression via the EGFR-ERK1/2 pathway. Brain Res. (2014) 1573:17–26. 10.1016/j.brainres.2014.05.022 24854121

[84] Kealy J Greene C Campbell M. Blood-brain barrier regulation in psychiatric disorders. Neurosci Lett. (2020) 726:133664. 10.1016/j.neulet.2018.06.033 29966749

[85] Villasenor R Lampe J Schwaninger M Collin L. Intracellular transport and regulation of transcytosis across the blood-brain barrier. Cell Mol Life Sci. (2019) 76:1081–92. 10.1007/s00018-018-2982-x 30523362 PMC6513804

[86] Stewart PA. Endothelial vesicles in the blood-brain barrier: are they related to permeability? Cell Mol Neurobiol. (2000) 20:149–63. 10696507 10.1023/A:1007026504843 PMC11537538

[87] Jasmin JF Malhotra S Singh DM Mercier I Rosenbaum DM Lisanti MP. Caveolin-1 deficiency increases cerebral ischemic injury. Circ Res. (2007) 100:721–9. 10.1161/01.RES.0000260180.42709.29 17293479

[88] Shin T Kim H Jin JK Moon C Ahn M Tanuma N. Expression of caveolin-1,−2, and−3 in the spinal cords of Lewis rats with experimental autoimmune encephalomyelitis. J Neuroimmunol. (2005) 165:11–20. 10.1016/j.jneuroim.2005.03.019 15925413

[89] Daneman R Engelhardt B. Brain barriers in health and disease. Neurobiol Dis. (2017) 107:1–3. 10.1016/j.nbd.2017.05.008 28552387

[90] Armulik A Genove G Betsholtz C. Pericytes: developmental, physiological, and pathological perspectives, problems, and promises. Dev Cell. (2011) 21:193–215. 10.1016/j.devcel.2011.07.001 21839917

[91] Cheslow L Alvarez JI. Glial-endothelial crosstalk regulates blood-brain barrier function. Curr Opin Pharmacol. (2016) 26:39–46. 10.1016/j.coph.2015.09.010 26480201

[92] Daneman R Zhou L Kebede AA Barres BA. Pericytes are required for blood-brain barrier integrity during embryogenesis. Nature. (2010) 468:562–6. 10.1038/nature09513 20944625 PMC3241506

[93] Armulik A Genove G Mae M Nisancioglu MH Wallgard E Niaudet C. Pericytes regulate the blood-brain barrier. Nature. (2010) 468:557–61. 10.1038/nature09522 20944627

[94] Bell RD Winkler EA Sagare AP Singh I LaRue B Deane R. Pericytes control key neurovascular functions and neuronal phenotype in the adult brain and during brain aging. Neuron. (2010) 68:409–27. 10.1016/j.neuron.2010.09.043 21040844 PMC3056408

[95] Park DY Lee J Kim J Kim K Hong S Han S. Plastic roles of pericytes in the blood-retinal barrier. Nat Commun. (2017) 8:15296. 10.1038/ncomms15296 28508859 PMC5440855

[96] Wang S Cao C Chen Z Bankaitis V Tzima E Sheibani N. Pericytes regulate vascular basement membrane remodeling and govern neutrophil extravasation during inflammation. PLoS ONE. (2012) 7:e45499. 10.1371/journal.pone.0045499 23029055 PMC3448630

[97] Ayloo S Lazo CG Sun S Zhang W Cui B Gu C. Pericyte-to-endothelial cell signaling via vitronectin-integrin regulates blood-CNS barrier. Neuron. (2022) 110:1641–55. 10.1016/j.neuron.2022.02.017 35294899 PMC9119930

[98] Wu L Ye Z Pan Y Li X Fu X Zhang B. Vascular endothelial growth factor aggravates cerebral ischemia and reperfusion-induced blood-brain-barrier disruption through regulating LOC102640519/HOXC13/ZO-1 signaling. Exp Cell Res. (2018) 369:275–83. 10.1016/j.yexcr.2018.05.029 29842876

[99] Methia N Andre P Hafezi-Moghadam A Economopoulos M Thomas KL Wagner DD. ApoE deficiency compromises the blood brain barrier especially after injury. Mol Med. (2001) 7:810–5. 10.1007/BF03401973 11844869 PMC1950012

[100] Abbott NJ. Inflammatory mediators and modulation of blood-brain barrier permeability. Cell Mol Neurobiol. (2000) 20:131–47. 10696506 10.1023/A:1007074420772 PMC11537513

[101] Xing G Zhao T Zhang X Li H Li X Cui P. Astrocytic sonic hedgehog alleviates intracerebral hemorrhagic brain injury via modulation of blood-brain barrier integrity. Front Cell Neurosci. (2020) 14:575690. 10.3389/fncel.2020.575690 33343302 PMC7747855

[102] Wang Y Jin S Sonobe Y Cheng Y Horiuchi H Parajuli B. Interleukin-1beta induces blood-brain barrier disruption by downregulating Sonic hedgehog in astrocytes. PLoS ONE. (2014) 9:e110024. 10.1371/journal.pone.0110024 25313834 PMC4196962

[103] Ralay RH Hodge JN Choi N Wainwright MS. Albumin induces upregulation of matrix metalloproteinase-9 in astrocytes via MAPK and reactive oxygen species-dependent pathways. J Neuroinflammation. (2012) 9:68. 10.1186/1742-2094-9-68 22507553 PMC3419618

[104] Garaschuk O Verkhratsky A. Physiology of microglia. Methods Mol Biol. (2019) 2034:27–40. 10.1007/978-1-4939-9658-2_3 31392675

[105] Bachiller S Jimenez-Ferrer I Paulus A Yang Y Swanberg M Deierborg T. Microglia in neurological diseases: a road map to brain-disease dependent-inflammatory response. Front Cell Neurosci. (2018) 12:488. 10.3389/fncel.2018.00488 30618635 PMC6305407

[106] Fantin A Vieira JM Gestri G Denti L Schwarz Q Prykhozhij S. Tissue macrophages act as cellular chaperones for vascular anastomosis downstream of VEGF-mediated endothelial tip cell induction. Blood. (2010) 116:829–40. 10.1182/blood-2009-12-257832 20404134 PMC2938310

[107] Da FA Matias D Garcia C Amaral R Geraldo LH Freitas C. The impact of microglial activation on blood-brain barrier in brain diseases. Front Cell Neurosci. (2014) 8:362. 10.3389/fncel.2014.00362 25404894 PMC4217497

[108] Mehrabadi AR Korolainen MA Odero G Miller DW Kauppinen TM. Poly(ADP-ribose) polymerase-1 regulates microglia mediated decrease of endothelial tight junction integrity. Neurochem Int. (2017) 108:266–71. 10.1016/j.neuint.2017.04.014 28461173

[109] Tao-Cheng JH Nagy Z Brightman MW. Tight junctions of brain endothelium in vitro are enhanced by astroglia. J Neurosci. (1987) 7:3293–9. 10.1523/JNEUROSCI.07-10-03293.1987 3668629 PMC6569185

[110] Lu A Suofu Y Guan F Broderick JP Wagner KR Clark JF. Matrix metalloproteinase-2 deletions protect against hemorrhagic transformation after 1 h of cerebral ischemia and 23 h of reperfusion. Neuroscience. (2013) 253:361–7. 10.1016/j.neuroscience.2013.08.068 24035828 PMC3827875

[111] Yang S Chen Y Deng X Jiang W Li B Fu Z. Hemoglobin-induced nitric oxide synthase overexpression and nitric oxide production contribute to blood-brain barrier disruption in the rat. J Mol Neurosci. (2013) 51:352–63. 10.1007/s12031-013-9990-y 23494638

[112] Alluri H Stagg HW Wilson RL Clayton RP Sawant DA Koneru M. Reactive oxygen species-caspase-3 relationship in mediating blood-brain barrier endothelial cell hyperpermeability following oxygen-glucose deprivation and reoxygenation. Microcirculation. (2014) 21:187–95. 10.1111/micc.12110 24372803

[113] Gibson CL Srivastava K Sprigg N Bath PM Bayraktutan U. Inhibition of Rho-kinase protects cerebral barrier from ischaemia-evoked injury through modulations of endothelial cell oxidative stress and tight junctions. J Neurochem. (2014) 129:816–26. 10.1111/jnc.12681 24528233

[114] Merlini M Meyer EP Ulmann-Schuler A Nitsch RM. Vascular beta-amyloid and early astrocyte alterations impair cerebrovascular function and cerebral metabolism in transgenic arcAbeta mice. Acta Neuropathol. (2011) 122:293–311. 10.1007/s00401-011-0834-y 21688176 PMC3168476

[115] Winkler EA Nishida Y Sagare AP Rege SV Bell RD Perlmutter D. GLUT1 reductions exacerbate Alzheimer's disease vasculo-neuronal dysfunction and degeneration. Nat Neurosci. (2015) 18:521–30. 10.1038/nn.3966 25730668 PMC4734893

[116] Plumb J McQuaid S Mirakhur M Kirk J. Abnormal endothelial tight junctions in active lesions and normal-appearing white matter in multiple sclerosis. Brain Pathol. (2002) 12:154–69. 10.1111/j.1750-3639.2002.tb00430.x 11958369 PMC8095734

[117] Kirk J Plumb J Mirakhur M McQuaid S. Tight junctional abnormality in multiple sclerosis white matter affects all calibres of vessel and is associated with blood-brain barrier leakage and active demyelination. J Pathol. (2003) 201:319–27. 10.1002/path.1434 14517850

[118] Leech S Kirk J Plumb J McQuaid S. Persistent endothelial abnormalities and blood-brain barrier leak in primary and secondary progressive multiple sclerosis. Neuropathol Appl Neurobiol. (2007) 33:86–98. 10.1111/j.1365-2990.2006.00781.x 17239011

[119] Nishihara H Perriot S Gastfriend BD Steinfort M Cibien C Soldati S. Intrinsic blood-brain barrier dysfunction contributes to multiple sclerosis pathogenesis. Brain. (2022) 145:4334–48. 10.1093/brain/awac019 35085379 PMC10200307

[120] Brown KA. Factors modifying the migration of lymphocytes across the blood-brain barrier. Int Immunopharmacol. (2001) 1:2043–62. 10.1016/S1567-5769(01)00129-1 11710535

[121] Alvarez JI Cayrol R Prat A. Disruption of central nervous system barriers in multiple sclerosis. Biochim Biophys Acta. (2011) 1812:252–64. 10.1016/j.bbadis.2010.06.017 20619340

[122] Alvarez JI Saint-Laurent O Godschalk A Terouz S Briels C Larouche S. Focal disturbances in the blood-brain barrier are associated with formation of neuroinflammatory lesions. Neurobiol Dis. (2015) 74:14–24. 10.1016/j.nbd.2014.09.016 25448765

[123] Sobel RA Mitchell ME Fondren G. Intercellular adhesion molecule-1 (ICAM-1) in cellular immune reactions in the human central nervous system. Am J Pathol. (1990) 136:1309–16. 1972610 PMC1877574

[124] Minten C Alt C Gentner M Frei E Deutsch U Lyck R. DARC shuttles inflammatory chemokines across the blood-brain barrier during autoimmune central nervous system inflammation. Brain. (2014) 137:1454–69. 10.1093/brain/awu045 24625696 PMC3999718

[125] Marchetti L Francisco D Soldati S Haghayegh JN Barcos S Gruber I. ACKR1 favors transcellular over paracellular T-cell diapedesis across the blood-brain barrier in neuroinflammation *in vitro*. Eur J Immunol. (2022) 52:161–77. 10.1002/eji.202149238 34524684 PMC9293480

[126] Baker AH Edwards DR Murphy G. Metalloproteinase inhibitors: biological actions and therapeutic opportunities. J Cell Sci. (2002) 115:3719–27. 10.1242/jcs.00063 12235282

[127] Vatansever S Schlessinger A Wacker D Kaniskan HU Jin J Zhou MM. Artificial intelligence and machine learning-aided drug discovery in central nervous system diseases: State-of-the-arts and future directions. Med Res Rev. (2021) 41:1427–73. 10.1002/med.21764 33295676 PMC8043990

[128] Ghose AK Viswanadhan VN Wendoloski JJ A. knowledge-based approach in designing combinatorial or medicinal chemistry libraries for drug discovery. 1 A qualitative and quantitative characterization of known drug databases. J Comb Chem. (1999) 1:55–68. 10.1021/cc9800071 10746014

[129] Pardridge WM. The blood-brain barrier: bottleneck in brain drug development. NeuroRx. (2005) 2:3–14. 10.1602/neurorx.2.1.3 15717053 PMC539316

[130] Antinori A Cingolani A Giancola ML Forbici F De Luca A Perno CF. Clinical implications of HIV-1 drug resistance in the neurological compartment. Scand J Infect Dis Suppl. (2003) 106:41–4. 10.1080/03008870310009650 15000582

[131] Sweeney MD Zhao Z Montagne A Nelson AR Zlokovic BV. Blood-brain barrier: from physiology to disease and back. Physiol Rev. (2019) 99:21–78. 10.1152/physrev.00050.2017 30280653 PMC6335099

[132] Sharp CD Hines I Houghton J Warren A Jackson TT Jawahar A. Glutamate causes a loss in human cerebral endothelial barrier integrity through activation of NMDA receptor. Am J Physiol Heart Circ Physiol. (2003) 285:H2592–8. 10.1152/ajpheart.00520.2003 12893641

[133] Yu Y Wu Y Wei J Huang F Mao F Nong W. NMDA mediates disruption of blood-brain barrier permeability via Rho/ROCK signaling pathway. Neurochem Int. (2022) 154:105278. 10.1016/j.neuint.2022.105278 35017026

[134] Anfray A Drieu A Hingot V Hommet Y Yetim M Rubio M. Circulating tPA contributes to neurovascular coupling by a mechanism involving the endothelial NMDA receptors. J Cereb Blood Flow Metab. (2020) 40:2038–54. 10.1177/0271678X19883599 31665952 PMC7786842

[135] Chen JT Chen TG Chang YC Chen CY Chen RM. Roles of NMDARs in maintenance of the mouse cerebrovascular endothelial cell-constructed tight junction barrier. Toxicology. (2016) 339:40–50. 10.1016/j.tox.2015.11.006 26655082

[136] Mao F Huang F Nong W Lao D Gong Z Huang W. N-methyl-D-aspartic acid increases tight junction protein destruction in brain endothelial cell via caveolin-1-associated ERK1/2 signaling. Toxicology. (2022) 470:153139. 10.1016/j.tox.2022.153139 35257817

[137] Kim KS Jeon MT Kim ES Lee CH Kim DG. Activation of NMDA receptors in brain endothelial cells increases transcellular permeability. Fluids Barriers CNS. (2022) 19:70. 10.1186/s12987-022-00364-6 36068542 PMC9450318

[138] Dzamba D Honsa P Anderova M NMDA. Receptors in Glial cells: pending questions. Curr Neuropharmacol. (2013) 11:250–62. 10.2174/1570159X11311030002 24179462 PMC3648778

[139] Verkhratsky A Chvatal A NMDA. Receptors in astrocytes. Neurochem Res. (2020) 45:122–33. 10.1007/s11064-019-02750-3 30767094

[140] Palygin O Lalo U Verkhratsky A Pankratov Y. Ionotropic NMDA and P2X1/5 receptors mediate synaptically induced Ca2+ signalling in cortical astrocytes. Cell Calcium. (2010) 48:225–31. 10.1016/j.ceca.2010.09.004 20926134

[141] Lalo U Pankratov Y Kirchhoff F North RA Verkhratsky A NMDA. receptors mediate neuron-to-glia signaling in mouse cortical astrocytes. J Neurosci. (2006) 26:2673–83. 10.1523/JNEUROSCI.4689-05.2006 16525046 PMC6675155

[142] Gottlieb M Matute C. Expression of ionotropic glutamate receptor subunits in glial cells of the hippocampal CA1 area following transient forebrain ischemia. J Cereb Blood Flow Metab. (1997) 17:290–300. 10.1097/00004647-199703000-00006 9119902

[143] Suhs KW Gudi V Eckermann N Fairless R Pul R Skripuletz T. Cytokine regulation by modulation of the NMDA receptor on astrocytes. Neurosci Lett. (2016) 629:227–33. 10.1016/j.neulet.2016.07.016 27423317

[144] Saab AS Tzvetavona ID Trevisiol A Baltan S Dibaj P Kusch K. Oligodendroglial NMDA Receptors Regulate Glucose Import and Axonal Energy Metabolism. Neuron. (2016) 91:119–32. 10.1016/j.neuron.2016.05.016 27292539 PMC9084537

[145] Traynelis SF Wollmuth LP McBain CJ Menniti FS Vance KM Ogden KK. Glutamate receptor ion channels: structure, regulation, and function. Pharmacol Rev. (2010) 62:405–96. 10.1124/pr.109.002451 20716669 PMC2964903

[146] Karadottir R Cavelier P Bergersen LH Attwell D NMDA. receptors are expressed in oligodendrocytes and activated in ischaemia. Nature. (2005) 438:1162–6. 10.1038/nature04302 16372011 PMC1416283

[147] Matute C Palma A Serrano-Regal MP Maudes E Barman S Sanchez-Gomez MV. N-methyl-D-aspartate receptor antibodies in autoimmune encephalopathy alter oligodendrocyte function. Ann Neurol. (2020) 87:670–6. 10.1002/ana.25699 32052483

[148] Cao N Yao ZX. Oligodendrocyte N-methyl-D-aspartate receptor signaling: insights into its functions. Mol Neurobiol. (2013) 47:845–56. 10.1007/s12035-013-8408-8 23345133

[149] Dahm L Ott C Steiner J Stepniak B Teegen B Saschenbrecker S. Seroprevalence of autoantibodies against brain antigens in health and disease. Ann Neurol. (2014) 76:82–94. 10.1002/ana.24189 24853231

[150] Pan H Oliveira B Saher G Dere E Tapken D Mitjans M. Uncoupling the widespread occurrence of anti-NMDAR1 autoantibodies from neuropsychiatric disease in a novel autoimmune model. Mol Psychiatr. (2019) 24:1489–501. 10.1038/s41380-017-0011-3 29426955 PMC6756099

[151] Gresa-Arribas N Titulaer MJ Torrents A Aguilar E McCracken L Leypoldt F. Antibody titres at diagnosis and during follow-up of anti-NMDA receptor encephalitis: a retrospective study. Lancet Neurol. (2014) 13:167–77. 10.1016/S1474-4422(13)70282-5 24360484 PMC4006368

[152] Wang Y Miao A Shi Y Ge J Wang L Yu C. Influencing electroclinical features and prognostic factors in patients with anti-NMDAR encephalitis: a cohort follow-up study in Chinese patients. Sci Rep. (2020) 10:10753. 10.1038/s41598-020-67485-6 32612192 PMC7329850

[153] Martinez-Hernandez E Horvath J Shiloh-Malawsky Y Sangha N Martinez-Lage M Dalmau J. Analysis of complement and plasma cells in the brain of patients with anti-NMDAR encephalitis. Neurology. (2011) 77:589–93. 10.1212/WNL.0b013e318228c136 21795662 PMC3149153

[154] Yu Y Wu Y Cao X Li J Liao X Wei J. The clinical features and prognosis of anti-NMDAR encephalitis depends on blood brain barrier integrity. Mult Scler Relat Disord. (2021) 47:102604. 10.1016/j.msard.2020.102604 33130468

[155] Ding Y Yang C Zhou Z Peng Y Chen J Pan S. Clinical significance of soluble adhesion molecules in anti-NMDAR encephalitis patients. Ann Clin Transl Neur. (2019) 6:945–53. 10.1002/acn3.740 31139692 PMC6529932

[156] Li Q Chen J Yin M Zhao J Lu F Wang Z. High level of soluble CD146 in cerebrospinal fluid might be a biomarker of severity of anti-N-methyl-D-aspartate receptor encephalitis. Front Immunol. (2021) 12:424. 10.3389/fimmu.2021.680424 34220828 PMC8245058

[157] Garcia-Serra A Radosevic M Pupak A Brito V Rios J Aguilar E. Placental transfer of NMDAR antibodies causes reversible alterations in mice. Neurol Neuroimmunol Neuroinflamm. (2021) 8:e915. 10.1212/NXI.0000000000000915 33172961 PMC7713722

[158] Zerche M Weissenborn K Ott C Dere E Asif AR Worthmann H. Preexisting serum autoantibodies against the NMDAR subunit NR1 modulate evolution of lesion size in acute ischemic stroke. STROKE. (2015) 46:1180–6. 10.1161/STROKEAHA.114.008323 25765725

[159] Gong Z Lao D Wu Y Li T Lv S Mo X. Inhibiting PI3K/Akt-signaling pathway improves neurobehavior changes in anti-NMDAR encephalitis mice by ameliorating blood-brain barrier disruption and neuronal damage. Cell Mol Neurobiol. (2023) 43:3623–37. 10.1007/s10571-023-01371-3 37314618 PMC10477152

[160] Castillo-Gomez E Kastner A Steiner J Schneider A Hettling B Poggi G. The brain as immunoprecipitator of serum autoantibodies against N-Methyl-D-aspartate receptor subunit NR1. Ann Neurol. (2016) 79:144–51. 10.1002/ana.24545 26505629

[161] Kreye J Wenke NK Chayka M Leubner J Murugan R Maier N. Human cerebrospinal fluid monoclonal N-methyl-D-aspartate receptor autoantibodies are sufficient for encephalitis pathogenesis. Brain. (2016) 139:2641–52. 10.1093/brain/aww208 27543972

[162] Li LY Kreye J Burek M Cordero-Gomez C Barthel PC Sanchez-Sendin E. Brain blood vessel autoantibodies in patients with NMDA and GABA(A) receptor encephalitis: identification of unconventional Myosin-X as target antigen. Front Cell Neurosci. (2023) 17:1077204. 10.3389/fncel.2023.1077204 36794262 PMC9922905

